# TRANSPARENT TESTA GLABRA1, a Key Regulator in Plants with Multiple Roles and Multiple Function Mechanisms

**DOI:** 10.3390/ijms21144881

**Published:** 2020-07-10

**Authors:** Hainan Tian, Shucai Wang

**Affiliations:** 1 Laboratory of Plant Molecular Genetics & Crop Gene Editing, School of Life Sciences, Linyi University, Linyi 276000, China; tianhainan2012@gmail.com; 2 Key Laboratory of Molecular Epigenetics of MOE, Northeast Normal University, Changchun 130024, China

**Keywords:** TTG1, MBW complex, cell fate determination, flavonoid biosynthesis, seed coat mucilage production, stress response, *Arabidopsis thaliana*

## Abstract

TRANSPARENT TESTA GLABRA1 (TTG1) is a WD40 repeat protein. The phenotypes caused by loss-of-function of *TTG1* were observed about half a century ago, but the *TTG1* gene was identified only about twenty years ago. Since then, TTG1 has been found to be a plant-specific regulator with multiple roles and multiple functional mechanisms. TTG1 is involved in the regulation of cell fate determination, secondary metabolisms, accumulation of seed storage reserves, plant responses to biotic and abiotic stresses, and flowering time in plants. In some processes, TTG1 may directly or indirectly regulate the expression of downstream target genes via forming transcription activator complexes with R2R3 MYB and bHLH transcription factors. Whereas in other processes, TTG1 may function alone or interact with other proteins to regulate downstream target genes. On the other hand, the studies on the regulation of TTG1 are very limited. So far, only the B3-domain family transcription factor FUSCA3 (FUS3) has been found to regulate the expression of *TTG1*, phosphorylation of TTG1 affects its interaction with bHLH transcription factor TT2, and TTG1 proteins can be targeted for degradation by the 26S proteasome. Here, we provide an overview of TTG1, including the identification of TTG1, the functions of TTG1, the possible function mechanisms of TTG1, and the regulation of TTG1. We also proposed potential research directions that may shed new light on the regulation and functional mechanisms of TTG1 in plants.

## 1. Introduction

The phenotypes of *Arabidopsis ttg* (*transparent testa*, *glabra*) mutants were first reported about 50 years ago [1,2]. It was then proposed that the *TRANSPARENT TESTA GLABRA* (*TTG*) locus has pleiotropic roles in the regulation of trichome initiation, anthocyanin biosynthesis, and seed coat mucilage biosynthesis in *Arabidopsis* [3]. The *TTG* locus was mapped to chromosome 5 about 40 years ago [4]. About 30 years ago, it was proposed that TTG1 might encode an MYC transcription factor [5]. However, *TTG1* gene was finally cloned about 20 years ago and was found to encode a WD40 repeat protein [6], a member of the WD40 repeat protein family, one of the largest protein families widely distributed in all eukaryotic cells. So far, more than 200 WD40 repeat proteins have been predicted in plants [7,8], and they are involved in the regulation of multiple processes in plants, such as signaling transduction, cell cycling, chromatin modification, transcriptional regulation, and RNA processing [7,9].

Accumulated evidence shows that, in addition to trichome initiation, anthocyanin biosynthesis, and seed coat mucilage production, TTG1 is also involved in the regulation of root hair formation [10], stomata development [11], seed development, post-embryonic development [3,6,12,13,14,15,16,17], proanthocyanidins (PAs) biosynthesis [3,13,17,18,19,20], accumulation of seed storage reserves such as fatty acids and proteins during the seed maturation process [15,16], as well as biotic and abiotic stress responses in plants [21,22,23].

In recent years, remarkable achievements have been made in understanding the roles and function mechanisms of TTG1 in regulating epidermal cell fate determination and secondary metabolism in *Arabidopsis* and other plants [10,11,12,13,14,15,16,17,18,19,20]. It has been proposed that TTG1 can interact with different R2R3 MYB and basic helix-loop-helix (bHLH) transcription factors to form multiple MYB-bHLH-WD40 (MBW) activator complexes to regulate the expression of downstream genes, thereby regulating cell fate determination including trichome initiation and root hair formation, and secondary metabolism including flavonoid biosynthesis and seed coat mucilage production [24,25,26,27,28,29]. For example, TTG1 regulates trichome initiation by forming MBW activator complexes with the R2R3 MYB transcription factor GLABRA1 (GL1), and the bHLH transcription factor GLABRA3 (GL3) or ENHANCER OF GLABRA3 (EGL3) to activate the expression of *GLABRA2* (*GL2*) [25,30]. The same MBW complexes can also activate the expression of some *R3 MYB* genes. Whereas R3 MYBs can compete with GL1 for the binding of GL3 or EGL3, thus preventing the formation of the MBW activator complexes, resulting in inhibition of trichome initiation [25,26,27,31]. TTG1 regulates root hair formation by forming MBW complexes with the R2R3 MYB transcription factor WERWOLF (WER) and the bHLH transcription factor GL3 or EGL3 to activate the expression of *GL2* [27,32,33]. TTG1 regulates flavonoid biosynthesis by forming MBW complexes with the R2R3 MYB transcription factors PRODUCTION OF ANTHOCYANIN PIGMENT 1 (PAP1), PAP2, MYB113, MYB114 or TRANSPARENT TESTA 2 (TT2) and the bHLH transcription factors TT8, GL3 or EGL3 to regulate the expression of the late biosynthesis genes in the flavonoid biosynthesis pathway [24,26,28,34,35,36,37,38].

Considering that some previous reviews have covered some aspects of the functions and function mechanisms of TTG1, for example, in the regulation of flavonoid biosynthesis [39,40] and evolution of cellular diversity as a component of MBW complexes [41]. We provide here an overview of TTG1, including the summary of its functions and possible functional mechanisms in regulating epidermal cell fate determination and secondary metabolism with an emphasis on recent progress, but with a specific focus on the identification history of TTG1, its functions that may not require the formation of the MBW complexes, the functions of TTG1 orthologs in other plants, and perspectives on potential future research directions.

## 2. Identification of TTG1

The *Arabidopsis* mutants showing a phenotype of yellow seeds and the absence of leaf trichomes were first reported in 1971 [1]. In 1978, the name *ttg* was assigned to these mutants [2]. In 1981, Koornneef found that the *ttg* mutants showed a pleiotropic phenotype including glabrous leaves, a transparent testa seed coat, reduced anthocyanin accumulation, and seed coat mucilage production [3]. In 1980, the gene *TTG1* was found to be located on chromosome 5 and is closely linked to *ms* [42]. In 1983, Koornneef et al. mapped the *TTG1* locus between the molecular marker *MS1* and *GA3* [4]. In 1999, Walkers et al. finally cloned *TTG1* gene by using positional cloning [6]. They generated two sets of recombinants between *ttg1-1* and the flanking genetic markers *MS1* and *GA3*, and performed RFLP (restriction fragment length polymorphisms) analysis by using a variety of probes. A total of 439 recombinants were subjected to large-scale mapping, and EG20H2, a clone from an *Arabidopsis* genomic yeast artificial chromosome (YAC) library [43], was found to cover the *TTG1* locus. Higher-resolution mapping of the region covered by EG20H2 showed that, when probed with g4556, a clone from an *Arabidopsis* genomic cosmid library [44], only a single recombinant could separate from *ttg1-1*, which suggested that the g4556 was very close to the *TTG1* locus. The g4556 and YAC EG20H2 clones were then used to screen an *Arabidopsis* genomic lambda (λ) library [45], and several λ clones were obtained. DNA probing with the obtained λ clones showed that the deletion of the genomic fragment in λ8 could account for the phenotype of *ttg1-13*, a fast-neutron bombardment *ttg* mutant [46]. Complementation of the *ttg1-1* mutant phenotype with entire and partial genomic fragments in λ8 confirmed that it contained the *TTG1* locus, and sequence analysis showed that *TTG1* encodes a WD40 repeat protein with high amino acid sequence identity and similarity to AN1 from petunia [6,47].

TTG1 has four WD40 repeat motifs [4], therefore, it is a member of the WD40 protein family. WD40 proteins are one of the largest regulatory protein families conserved in all eukaryotic cells [9], and the only conserved feature of WD40 proteins is the presence of the WD40 motifs, which typically contain several copies of WD40 repeats, with each repeat containing 44–60 amino acid residues, with a glycine-histidine (GH) dipeptide at the N-terminal and Trp-Asp (WD) residues at the C-terminal [9]. WD40 proteins are able to provide a platform for interactions with other proteins and are involved in the regulation of growth and development, as well as some cellular functions such as signal transduction and transcriptional regulation [7,8,9].

After being summarized previously [39], a few more *Arabidopsis ttg1* mutants were identified, and a complete list of *Arabidopsis ttg1* mutants identified so far are presented in Table 1. By characterizing these mutants, it has been found that TTG1 regulates multiple aspects of plant growth and development, secondary metabolism, flowering time, and plant responses to environmental stresses.

## 3. Functions and Function Mechanisms of TTG1

**Figure 1:**
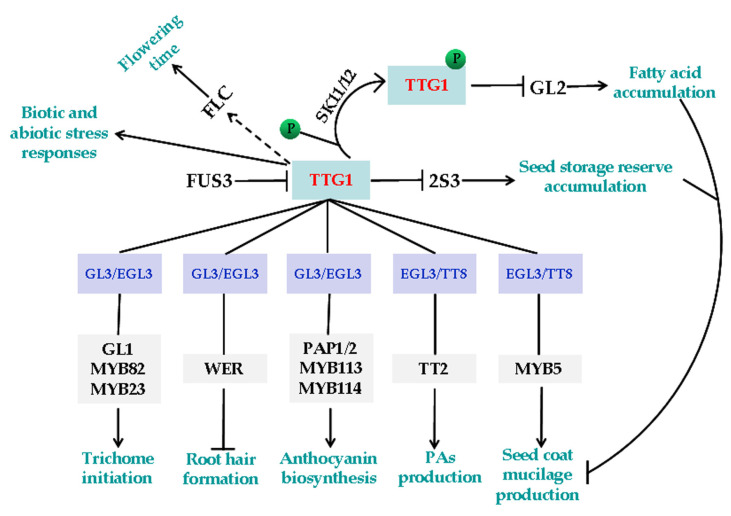
The functions of TTG1. TTG1 regulates cell fate determination and secondary metabolism by forming MBW complexes with specific R2R3 MYB and bHLH transcription factors. The TTG1-GL3/EGL3-GL1/MYB23/MYB82 complexes regulate trichome initiation [25,30,60,61], the TTG1-GL3/EGL3-WER complexes regulate root hair formation [27,33], the TTG1-GL3/EGL3-PAP1/2/MYB113/114 complexes regulate anthocyanin biosynthesis [24,34,36], the TTG1-EGL3/TT8-TT2 complexes regulate proanthocyanidins (PAs) biosynthesis [24,28,34], and the TTG1-EGL3/TT8-MYB5 complexes regulate seed coat mucilage production [24]. TTG1 compromises the accumulation of seed storage reserves through inhibiting 2S3, and FUS3 can directly suppress the expression of *TTG1*. SK11/12 can phosphorylate TTG1, therefore inhibit *GL2* expression, and affect fatty acid accumulation. TTG1 is also involved in regulating flowering, as well as biotic and abiotic stress responses.

TTG1 is a plant-specific WD40 protein widespread in angiosperms but not in gymnosperms and early evolved plants [58]. TTG1 has similar functions in different plant species. At least some of its functions including the regulation of trichome initiation, root hair formation, flavonoids biosynthesis, and seed coat mucilage production are achieved via interacting with specific R2R3 MYB and bHLH transcription factors to form different MBW complexes (Figure 1). However, it should be noted that there is a study showing that TTG1 and GL1 compete for binding of GL3 to form TTG1-GL3 or GL1-GL3 dimers, rather than simultaneously bind to GL3 to form TTG1-GL3-GL1 trimeric complex, the interaction of GL3 with GL1 and TTG1 can be suppressed by additional TTG1 and GL1 protein, and the competition/suppression occurs in a dosage-dependent manner [59].

### 3.1. Regulation of Epidermal Cell Fate Determination

In *Arabidopsis*, TTG1 regulates the specification of several different types of epidermal cells including trichomes, root hairs, and stomata. The *ttg1-1* mutants do not produce trichomes [3,6], suggesting that TTG1 is involved in the regulation of trichome initiation. Further studies indicate that TTG1 regulates trichome initiation via associating with the R2R3 MYB transcription factor GL1 [62] and the bHLH transcription factors GL3 or EGL3 [34,63] to form MBW activator complexes (Figure 1), the complexes activate the expression of the downstream gene *GL2* to promote trichome initiation and development [32]. The TTG1-GL3/EGL3-GL1 complexes can also regulate the expression of several single *R3 MYB* genes, whereas R3 MYBs can move from the trichome cells to the neighboring cells, where they compete with GL1 for binding of GL3 or EGL3 to prevent the formation of the MBW complexes, resulting in inhibition of trichome initiation [25,26,27,31,64]. TTG1 can also interact with the *miR156*-targeted SQUAMOSA PROMOTER BINDING PROTEIN-LIKE (SPL) transcription factors SPL4 or SPL5, which affects the transcriptional activity of the MBW complexes and leads to inhibition of trichome initiation [65].

Different from trichome initiation, root hair formation follows a position-dependent manner. In wild-type *Arabidopsis*, hair cells (H cells; trichoblasts) can form root hairs, whereas hairless cells (N cells; atrichoblasts) cannot [66,67]. In the *ttg1-1* mutants, the number of root hairs is increased due to ectopic root hair formation in N cells [10]. However, a decrease in root hair formation was observed in *ttg1-23* and *ttg1-24*, two mutants with a single amino acid substitution in the TTG1 protein [51] (Table 1). Similar to the regulation of trichome initiation, TTG1 regulates root hair formation via forming MBW complexes, however, GL1 is replaced by another R2R3 MYB transcription factor, WER [68]. The TTG1-GL3/EGL3-WER complexes induce the expression of *GL2* and some *R3 MYBs*. Different from their inhibitory function in regulating trichome initiation, R3 MYBs can promote root hair formation. R3 MYBs can move from N cell to H cell, where they compete with WER for binding of GL3 or EGL3, therefore, preventing the formation of the MBW complexes, resulting in the promotion of root hair formation [60,69,70,71]. In the *ttg1-23* and *ttg1-24* mutants, the single amino acid substitution in TTG1 abolished its interaction with the bHLH transcription factor GL3/EGL3, therefore, differentially affected the expression of the MBW complex target genes in the mutants. These results demonstrate that TTG1 may be able to balance the target genes expression to enable the plant to produce a proper amount of different types of epidermal cells during the root development [51].

Unlike trichomes and root hairs, stomata are developed from precursor cells. The *ttg1-1* mutants produced more ectopic stomata on the hypocotyls, but stomata distribution in cotyledons and leaves remained unchanged [11]. As the *gl2* mutants also produced more ectopic stomata on the hypocotyls [11], TTG1 might regulate stomata development via regulating the expression of *GL2*. Previous studies showed that the TTG1 may function epistatic to GL2 in regulating root hair formation [10,32]. This could also be the case for TTG1 in regulating stomata development [11].

Available evidence shows that TTG1 orthologs in several other plants are also able to regulate epidermal cell fate determination. For example, transgenic *Arabidopsis* plants ectopically expressing the *Limonium bicolor TTG1* (*LbTTG1*) produced more trichomes and fewer root hairs [21]; expression of *TTG1* ortholog genes from other plant species including the *Gossypium hirsutum TTG1* (*GhTTG1*) and *GhTTG3*, the *Malus domestica TTG1* (*MdTTG1*), and the *maize PAC* restored trichome defect phenotype in the *ttg1* mutants [72,73,74]; the *Arabis alpina ttg1* (*aattg1*) mutants showed a glabrous phenotype and produced more root hairs [75]; expression of the *Cucumis sativus TTG1* (*CsTTG1*) in cucumber promoted fruit trichome and spine formation, whereas silencing of *CsTTG1* inhibited fruit spine initiation. However, TTG1 in other plants may use different mechanisms to regulate epidermal cell fate determination, as molecular and genetic analysis show that even though CsTTG1 acts in parallel to CsGL1 in regulating fruit trichome initiation, CsTTG1 can directly interact with CsGL1, whereas in *Arabidopsis*, TTG1 directly interacts GL3 but not GL1 [76].

### 3.2. Regulation of Flavonoid Biosynthesis

Flavonoids including proanthocyanidins (PAs) and anthocyanins are secondary metabolites in higher plants [19,34,35]. The *ttg1-1* mutants do not accumulate anthocyanins and can produce yellow seeds [3,6,18,24], suggesting that TTG1 is involved in the regulation of PA and anthocyanin biosynthesis. Similar to the regulation of cell fate determination, TTG1 in *Arabidopsis* regulates flavonoid biosynthesis via forming MBW complexes. TTG1 can form MBW complexes with the R2R3 MYB transcription factors PAP1, PAP2, MYB113, MYB114 or TT2, and the bHLH transcription factors GL3, EGL3 or TT8 [24,26,28,34,35,36,37,38,77,78]. It has been shown that different MBW complexes regulate the biosynthesis of different flavonoids. The TTG1-TT8/GL3-PAP1/PAP2/MYB113/MYB114 complexes regulate anthocyanin biosynthesis by activating the expression of late biosynthetic genes including *DIHYDROFLAVONOL 4-REDUCTASE* (*DFR*), *ANTHOCYANIDIN SYNTHASE* (*ANS*) and *UDP-GLUCOSE:FLAVONOID 3-O-GLUCOSYLTRANSFERASE* (*UF3GT*) in the anthocyanin biosynthetic pathway [36,78,79]. Whereas the TTG1-GL3/TT8–TT2 complexes regulate PA accumulation by activating the expression of *DFR*, *ANS*, *BANYULS* (*BAN*), *TT19*, and *TT12* [28,38,79]. Whole genome-wide identification studies of the TTG1-dependent MBW complex target genes found that the TTG1-dependent MBW complexes are able to bind directly to the promoters of *TTG2*, *TT8*, *FLAVANONE 3-HYDROXYLASE* (*F3′H*), *DFR*, and *ANS* genes to regulate their expression [80].

The TTG1 orthologs in other plant species have also been found to regulate flavonoid biosynthesis. For example, ectopic expression of the *Freesia hybrida TTG1* (*FhTTG1*) in the *ttg1-1* mutants partially restored the anthocyanin biosynthesis deficient phenotype [81]. Ectopic expression of *CsWD40*, a *TTG1* homolog gene in *Camellia sinensis* and tobacco increased anthocyanin accumulation [82]. *Salvia miltiorrhiza TTG1* (*SmTTG1*) is also involved in the regulation of anthocyanin accumulation [83]. It has been shown that FhTTG1 is able to interact with FhTT8L and FhGL3L [81], and CsWD40 is able to interact with CsGL3/CsTT8 and CsAN2/CsMYB5e [82], suggesting that TTG1 in these plants may use similar mechanisms as in *Arabidopsis* to regulate flavonoid biosynthesis. On the other hand, the bHLH transcription factor in *Arabidopsis* often functions as a bridge between MYB transcription factors and TTG1 [28,84,85]. However, in both bimolecular fluorescence complementation (BiFC) and yeast two-hybrid (Y2H) experiments, SmMYB111 interacted with SmTTG1 and SmbHLH51, but SmTTG1 did not interact with SmbHLH51 [82]. This suggests that SmMYB111 may function as a bridge between SmTTG1 and SmbHLH51 to form a SmTTG1-SmMYB111-SmbHLH51 complex in *S. miltiorrhiza*.

### 3.3. Regulation of Seed Coat Mucilage Production

In addition to the defects on cell fate determination and flavonoid biosynthesis, the *ttg1-1* mutants also have defects in seed coat mucilage production [3], suggesting that TTG1 is involved in the regulation of seed coat mucilage production. Similar to that in the regulation of cell fate determination and flavonoid biosynthesis, TTG1 regulates seed coat mucilage production via forming MBW complexes. TTG1 can form MBW complexes with the bHLH transcription factors TT8 or EGL3 and the R2R3 MYB transcription factors MYB5 or TT2 [24,86,87,88]. The MBW complexes can activate the expression of *TTG2* and *GL2* [89,90], GL2, in turn, activates the expression of *MUCILAGE MODIFIED 4* (*MUM4*), a mucilage biosynthesis gene, leading to the production of seed coat mucilage [90]. It should be noted that not all the MBW complex components play an equal role in regulating seed coat mucilage production, as an example, MYB5 plays a key role in the regulation of seed coat mucilage production, whereas TT2 has only a minor effect [38,91,92]. It should be also noted that some regulators such as APETALA2 (AP2), NAC-REGULATED SEED MORPHOLOGY1(NARS1), NARS2, MYB52, MYB61, and LUENIG HOMOLOG (LUH) can regulate seed coat mucilage production independent of TTG1 [17,29,93,94,95].

In addition to directly regulate seed coat mucilage production as described above, TTG1 can also indirectly regulate seed coat mucilage production via regulating fatty acid accumulation in seeds [15,96]. As the content of total proteins and fatty acids is increased in the *ttg1* mutant embryos, the dry weight of the *ttg1* mutant embryos is significantly increased compared to the wild-type plants. Molecular and genetic analysis indicated that TTG1 suppresses the accumulation of seed storage proteins partially through inhibiting the expression of the 2S albumin precursor gene *2S3*. On the other hand, *TTG1* is a direct target of the seed maturation master regulator FUSCA3 (FUS3), which can inhibit the expression of *TTG1* in developing seeds [15]. The SHAGGY-like kinase 11 (SK11) and SK12 are able to phosphorylate TTG1 at the serine 215, therefore preventing the interaction of TTG1 with TT2, leads to an increase in fatty acid biosynthesis in the embryo, but a decrease in seed coat mucilage production in the *sk11 sk12* mutants [16].

Ectopic expression of *Setaria italica TTG1* (*SiTTG1*) in the *ttg1-13* mutant successfully restored its phenotypes of a high content of fatty acids, transparent seed coat, storage protein contents, as well as seed coat mucilage defects, indicating that SiTTG1 has similar functions to TTG1 in regulating seed coat mucilage production and fatty acid accumulation [97].

### 3.4. Regulation of Flowering Time

Plant flowering is an important trait for the transition from the vegetative phase to the reproductive phase. In long-day conditions, the *ttg1* mutants flowered earlier and the *TTG1* over-expression plants flowered later than the wild-type plants [98], suggesting that TTG1 is involved in the regulation of flowering time. It is known that FLOWERING LOCUS C (FLC) can repress the expression of *FLOWERING LOCUS T* (*FT*) and *SUPPRESSOR OF OVEREXPRESSION OF CO 1* (*SOC1*) by binding to their promoter regions [99]. Paffendorf et al. found that TTG1 can activate the expression of *FLC* [98]. They also found that TTG1 may also regulate the circadian clock component genes. By screening yeast two-hybrid libraries, the author identified PSEUDO RESPONSE REGULATOR 5 (PRR5) and bHLH92 as TTG1 interactors, and they found that TTG1 is able to modulate bHLH92 localization [94].

It has previously been reported that the expression of *PRR5* can be directly regulated by LIGHT-REGULATED WD 1 (LWD1) [100]. Whereas, LWD1 and LWD2 are two closely related WD40 repeat proteins to TTG1, and they function redundantly in the photoperiodic pathway to regulate flowering time in *Arabidopsis*, as the *lwd1 lwd2* double mutants flowered early in long-day condition and the expression level of *FT* was increased in the mutants [101]. These results indicate that TTG1, LWD1, and LWD2 may function in the same pathway to regulate flowering time in plants. On the other hand, the *lwd1 lwd2 ttg1* triple mutants lack detectable circadian rhythms, indicating that TTG1 is a regulator of the circadian system [102]. However, TTG1 could rescue the phenotypes of *ttg1* and *lwd1 lwd2* mutants, but LWD1 and LWD2 could not rescue the epidermal defects of *ttg1* mutants, suggesting that subfunctionalization happened following the divergence of the TTG1 and LWD proteins in angiosperms [102].

### 3.5. Regulation of Biotic and Abiotic Stress Responses

Some available experimental evidence suggests that TTG1 is involved in the regulation of plant response to abiotic stresses. First, the seed germination rate of the *ttg1* mutants is lower than Col in response to NaCl treatment, and NaCl treatment also affects leaf and root development in the *ttg1* mutant seedlings, indicating that TTG1 is involved in the regulation of plant response to salt stress [21,97]. Second, the *ttg1* mutants are also sensitive to sucrose stresses [96]. Third, ectopic expression of *SiTTG1* completely restores the salt and sucrose sensitivity phenotypes observed in the *ttg1* mutants, indicating that SiTTG1 and TTG1 have a similar function in regulating plant response to salt and sucrose stresses [97]. Forth, ectopic expression of *LbTTG1* in *Arabidopsis* enhanced salt stress tolerance [21]. Under NaCl treatment, the transgenic plants accumulated less Na^+^ and malondialdehyde (MDA), but more K^+^, proline, and soluble sugar when compared with that in the wild-type plants, and an elevated expression level of salt-tolerance marker genes including *SALT OVERLY SENSITIVE 1* (*SOS1*), *SOS2*, *SOS3*, and *PYRPOLINE-5-CARBOXYLATE SYNTHASE 1* (*P5CS1*) was observed in the *LbTTG1* transgenic plants [21]. Fifth, ectopic expression of *TaWD40D*, the *Triticum aestivum TTG1* also enhanced salt and osmotic stress tolerance in *Arabidopsis* [22]. In addition, the expression of *CsWD40* is induced by ABA and sucrose treatments, indicating that *CsWD40* may be involved in the regulation of abiotic stress responses in tea plants [82].

Recently, it has been shown that SAR DEFICIENT 4 (SARD4), RECEPTOR LIKE PROTEIN (RLP3), RLP27, RLP26, and PATHOGENESIS-RELATED GENE 6 (PR6) regulate plant immunity in a TTG1-dependent manner [103]. It has also been reported that the TTG1 ortholog Tannin1 (Tan1) is responsible for pathogen-induced color variation in *Sorghum bicolor* [104], and *tobacco* TTG1 (NtTTG1) physically interacts with the oomycete-specific effector ParA1 to regulate plant immune responses, including reactive oxygen species (ROS) production and programmed cell death [23]. These experiments support that TTG1 plays a role in regulating plant response to biotic stresses.

### 3.6. Other Functions

In addition to the functions mentioned above, TTG1 may also have a few other functions. TTG1 can regulate wax ester biosynthesis by suppressing the expression of *LONG-CHAIN ACYL-COA SYNTHETASE* 3 (*LACS3*), *FATTY ACID REDUCTASE 6* (*FAR6*), *WSD1*, *LIPID TRANSFER PROTEIN 5* (*LTP5*), *LTP10* and *ATP-BINDING CASSETTE G23* (*ABCG23*) [103]. TTG1 can also regulate cutin biosynthesis by regulating the expression of *CYTOCHROME P450*, *FAMILY 96*, *SUBFAMILY A*, *POLYPEPTIDE 11* (*CYP96A11*), and *CUS4* [103]. The expression of several hormone modification genes including *UDT-DEPENDENT GLYCOSYLTRANSFERASE 75 D* (*UGT75D*), *BETA GLUCOSIDASE 18* (*BGLU18*), and *BRASSINOSTEROID INACTIVATOR 1* (*BIA1*) is regulated by TTG1, indicating that TTG1 may regulate hormone metabolism [103]. It has been reported that the *Salvia miltiorrhiza* MYB111 (SmMYB111) positively regulates the biosynthesis of phenolic acids, Sal B, and rosmarinic acid (RA) by forming the SmTTG1−SmbHLH51−SmMYB111 transcription complex, suggesting that TTG1 may play a role in regulating the biosynthesis of phenolic acids, Sal B, and RA [83].

## 4. Regulation of TTG1

### 4.1. Transcriptional Regulation

Studies on the transcriptional regulation of *TTG1* are very limited. So far, only the B3-domain family transcription factor FUS3 has been identified as a regulator of *TTG1*. Tsuchiya et al. found that the expression level of *TTG1* is increased in the embryos of the *fus3* mutants, and the *fus3* mutant phenotypes including defects in anthocyanin biosynthesis and seed storage proteins accumulation are recovered in the *fus3 ttg1* double mutant, suggesting that expression of *TTG1* is down-regulated by FUS3 [14]. By using a dexamethasone-inducible transgenic plant system, Chen et al. found that *TTG1* may be an immediate target of FUS3, by using ChIP-PCR and an electrophoretic mobility shift assay (EMSA), they found that the FUS3 can bind directly to the consensus binding site of B3-domain family transcription factors in the promoter region of *TTG1*, indicating that *TTG1* is a direct target of FUS3 [15].

### 4.2. Posttranscriptional Regulation

Protein degradation by the ubiquitin/26S proteasome system (UPS) pathway affects protein functions. Patra et al. demonstrated that the TTG1 proteins are short-lived and can be targeted by UPS for degradation [105]. They generated transgenic plants overexpressing FLAG targeted *TTG1* in the *ttg1* (Salk_104152) mutants and used anti-FLAG antibodies to examine TTG1 fusion protein in the transgenic plants. They found that the amount of the TTG1 fusion protein was depleted significantly when the seedlings were treated with cycloheximide (CHX), a protein synthesis inhibitor. Whereas depletion of TTG1 fusion protein was inhibited by combined treatment of CHX and MG132, a peptide aldehyde that can selectively inhibit the proteolytic activity of the 26S proteasome, suggesting that TTG1 can be targeted by UPS for degradation [105].

Protein phosphorylation can also affect protein functions. Both SK11 and SK12 and the GSK3-like kinase BRASSINOSTEROID-INSENSITIVE 2 (BIN2) have been shown to be able to phosphorylate TTG1. SK11 and SK12 can interact with TTG1 and phosphorylate TTG1 at serine 215, thus preventing the interaction of TTG1 with TT2, resulting in inhibition of the MBW complex formation [16]. Previous studies have shown that the GSK3-like kinases may be involved in BR-mediated root epidermal cell fate determination by acting upstream of the TTG1–GL3/EGL3–WER MBW complexes [106]. By using GST pull-down and BiFC to test the interaction between the MBW complex component proteins and the GSK3-like kinase BIN2, Cheng et al. found that BIN2 can interact with TTG1, and in vitro kinase assays confirmed that BIN2 could also phosphorylate TTG1 [107].

## 5. Challenges and Future Perspectives

As mentioned above, TTG1 has been found to regulate multiple aspects of plant growth and development, secondary metabolism, as well as plant response to environmental stimuli, and the functional mechanisms of TTG1 have also been extensively studied. Yet, more efforts are required to further elucidate the regulation and functional mechanisms of TTG1 in plants.

It has been shown that in some processes, such as trichome initiation, root-hairs formation, flavonoid biosynthesis, and seed coat mucilage production, TTG1 needs to interact with specific R2R3 MYB and bHLH transcription factors to form different MBW complexes to regulate the expression of downstream genes [24,25,26,27,28]. However, it has also been shown that GL1 and TTG1 may compete for binding with GL3 to form GL1-GL3 and GL3-TTG1 dimers [58], and dimers formed by an R2R3 MYB and a bHLH transcription factor are sufficient to activate the expression of *GL2* and some *R3 MYB* genes [108], ectopic expression of maize *R*, a bHLH transcription factor gene complemented all the phenotypes of *ttg1* mutants [5], and TT2 binds to GL2 promoter independent of TTG1, but in the absence of TT2, TTG1 can still bind to *GL2* promoter [16]. This evidence suggests that formation of the MBW complexes may not be necessary for TTG1 to regulate downstream gene expression. Considering that regulators other than bHLH transcription factors, such as PRR5 and ParA1, which are involved in the regulation of flowering time and plant immune responses, respectively [23,98], have been shown to be able to interact with TTG1, it will be of interest to examine if TTG1 may interact with other regulators to regulate the cell fate determination, flavonoid biosynthesis, and seed coat mucilage production. On the other hand, CsTTG1 interacts directly with CsGL1 to regulate fruit trichome initiation in *C. sativa* [76], and *SmTTG1* interacts with SmMYB111 to regulate phenolic acid biosynthesis complex in *S. miltiorrhiza* [83], it will be of interest to examine why different plants may use the different bridge proteins to form MBW complexes.

In addition, TTG1 interacts with different bHLH transcription factors to regulate specification of different cell types and different secondary metabolisms [24,25,26,27,28,29]. It has been shown that phosphorylation of TTG1 by SK11 and SK12 at the serine 215 is able to prevent the interaction of TTG1 with TT2, therefore, affecting fatty acids biosynthesis in embryo, mucilage, and flavonoid production in the seed coat [16], so it will be meaningful to detect whether the phosphorylation of TTG1 by SK11 and SK12 may also affect its interaction with other interactors involved in the regulation of cell fate determination, therefore, affecting its functions in regulating cell fate determination, or if different kinases may be involved in these processes.

Some experiments suggest the TTG1 may be required for the stabilization of the bHLH-MYB dimers, as the absence of TTG1 weakened the interaction between MYB and bHLH transcription factor in transfected protoplasts [28,63]. Consistent with this, it has been shown that the *ttg1-10*, a mutant with a point mutation in the 5′-*UTR* region showed a transparent testa seed coat phenotype, but anthocyanin synthesis and trichome initiation was largely unaffected, suggesting that reduced TTG1 protein level may have a big effect on the stabilization of the TT8-TT2 dimer, but not the PAP1/PAP2/GL1-GL3 dimers [46]. TTG1 proteins are short-lived and can be targeted for UPS degradation [105], but the ligases involved remain unknown. Identification of the ligases and the examination of how the degradation of TTG1 may affect the stability of the bHLH-MYB dimers will also help to understand the functional mechanisms of TTG1.

The regulation of *TTG1* expression also needs to be further studied. FUS3 has been identified as a negative regulator of *TTG1* [15]. Identification and characterization of positive regulators of *TTG1* may help further explore the functional mechanisms of TTG1. It should be noted that similar to the *fus3* mutant, the *leafy cotyledon 1* (*lec1*) and *lec2* mutants can produce cotyledon trichomes [109], LEC2 is also a B3-domain family transcription factor [110], and is able to interact with FUS3 [111], it is worthwhile to examine if LEC1 and LEC2 may also regulate the expression of *TTG1*.

## Figures and Tables

**Table 1:** The *Arabidopsis ttg1* mutants identified so far.

Allele	Origin	Mutation	Phenotype	References
*ttg1-1*	EMS	Q317-stop codon	transparent testa, glabra	[3,6]
*ttg1-9*	EMS	S282F	transparent testa, glabra	[6,48]
*ttg1-10*	EMS	g-a (5′UTR)	transparent testa, glabra (−)	[46]
*ttg1-11*	EMS	G149R	transparent testa, glabra	[46]
*ttg1-12*	EMS	G43R	transparent testa, glabra	[46]
*ttg1-13*	fast neutrons	genome deletion	transparent testa, glabra	[46]
*ttg1-15/16/17/18*	EMS	S310-stop codon	transparent testa, glabra	[6]
*ttg1-19*	EMS	W183-stop codon	transparent testa, glabra	[6]
*ttg1-20*	EMS	S30C, S310-stop codon	transparent testa, glabra	[6]
*ttg1-21*	T-DNA	insert in 5′UTR	transparent testa, NA	[49,50]
*ttg1-22*	T-DNA	insert in intron	transparent testa, NA	[49,50]
*ttg1-23*	EMS	S197F	transparent testa (−), glabra	[51]
*ttg1-24*	EMS	L339F	transparent testa (−), glabra (−)	[51]
*ttg1-23* (T)	T-DNA	fragment deletion	transparent testa, glabra	[52]
*ttg1-24* (T)	T-DNA	genome deletion	transparent testa, glabra	[51]
*ttg1-213*	NA	W183-stop codon	transparent testa, glabra	[53]
*urm23*	EMS	G302E	glabra (− −)	[53]
*ttg1* (*Est*)	EMS	S101F	transparent testa, glabra	[54]
*ttg1-P313*	T-DNA	Insertion (ND)	glabra	[55,56]
*ttg1-P416*	T-DNA	insert in intron	transparent testa, glabra	[55,56]
*ttg1-SK31268*	T-DNA	Insertion (ND)	transparent testa, glabra	[55,56]
*ttg1-SK41546*	T-DNA	insert in intron	transparent testa, glabra	[55,56]
*ttg1-21-CI*	CI	insert in 5′UTR	transparent testa, NA	[57]

Phenotypes from strong to weak: glabra > glabra (−) > glabra (− −); transparent testa > transparent testa (−). Est: Estland, EMS: Ethyl methanesulfonate, T-DNA: transfer-DNA, UTR: untranslated region, NA: no information available, *urm*: *unarmed*, CI: carbon ion irradiation, ND: position of insertion was not determined.
